# Efficacy of Dietary Intervention with Group Activities on Dietary Intakes, Frailty Status, and Working Memory: A Cluster-Randomized Controlled Trial in Community Strongholds

**DOI:** 10.3390/nu15081976

**Published:** 2023-04-19

**Authors:** Szu-Yun Wu, Yu-Yao Cheng, Hsing-Yi Chang, Pei-Hsuan Wang, I-Ching Hsieh, Nai-Hua Yeh, Kuo-Chin Huang, Wen-Harn Pan

**Affiliations:** 1 Institute of Biomedical Sciences, Academia Sinica, 128 Sec. 2, Academia Rd. Nankang, Taipei 115, Taiwan; s.wu@ibms.sinica.edu.tw (S.-Y.W.);; 2 Department of Health and Nutrition, Chia Nan University of Pharmacy and Science, No. 60, Sec. 1, Erren Rd., Rende Dist., Tainan City 717, Taiwan; 3 Institute of Population Health Sciences, National Health Research Institutes, 35, Keyan Road, Zhunan Town, Miaoli County 350, Taiwan; 4 Department of Family Medicine, National Taiwan University Hospital Hsin-Chu Branch, No. 2, Sec. 1, Shengyi Rd., Zhubei City, Hsin-Chu County 302, Taiwan; 5 Department of Food Nutrition and Health Biotechnology, Asia University, No. 500, Lioufeng Rd., Wufeng, Taichung 41354, Taiwan

**Keywords:** frailty, older adults, community strongholds, dietary intervention, group activities, working memory

## Abstract

Geriatric community centers often offer nutrition lectures to older adults. In order to make learning more interesting and pragmatic, we developed group activity sessions. This undertaking was tested for its efficacy in changes of frailty status and several other geriatric health parameters. A cluster-randomized controlled trial was conducted between September 2018 and December 2019 at 13 luncheon-providing community strongholds in Taipei, Taiwan. During the 3-month intervention period, 6 experimental strongholds received a weekly 1 h exercise workout and 1 h nutrition activities aiming at achieving the recommendations of the Taiwanese Daily Food Guide for elderlies; the other 7 received a weekly 1 h exercise workout and 1 h other activities. Dietary intakes and frailty status were the primary outcomes. Secondary outcomes included working memory and depression. The measurements were performed at baseline, 3 months, and 6 months. The nutrition intervention significantly reduced the intake of refined grains and roots (*p* = 0.003) and increased that of non-refined grains and roots (*p* = 0.008), dairy products (*p* < 0.0001), and seeds and nuts (at borderline, *p* = 0.080) at 3 months. Some, but not all, of these changes were maintained at 6 months. Performance improvements included the frailty status score (*p* = 0.036) and forward digit span (*p* = 0.004), a working memory parameter, at 3 months. Only the forward digit span remained improved (*p* = 0.007) at 6 months. The 3-month nutrition group activities combined with exercise sessions improved the frailty status and working memory more than exercise alone. The dietary and frailty improvements were accompanied by improved dietary intakes and advanced behavioral stages. However, the improved frailty status backslid after intervention ceased, suggesting that boosting activities are needed for maintaining the intervention effect.

## 1. Introduction

Taiwan has been an aged society since 2018 and will become a super-aged society in 2025 [1]. In the year of 2020, 10.3% of the older adults were aged above 85 years [1]. Taiwan’s government is implementing a 10-year Long-term Care Plan 2.0 [2]. Under this project, the government has established thousands of Geriatric Community Caring Strongholds island-wide to provide meals and learning programs for the social participation of older adults in the community [3]. This study is a result of a response to a request for proposal from the Taiwan Health Promotion Agency to design and evaluate the efficacy of fun group activities for improving nutrition knowledge and behaviors.

According to the literature and our previous analytical epidemiological studies [4,5,6,7], older adults with geriatric syndromes tend to have lower intakes of total energy, macronutrients, and multiple vitamins and minerals. The role of adequate protein and calories in preventing or reversing frailty and/or sarcopenia have been repeatedly confirmed from the findings of clinical trials, particularly when combined with muscle building and balance training [8]. However, experiments or trials are scarce for micronutrients. Nonetheless, evidence has accumulated to show that healthy dietary patterns tend to lower the risk of developing geriatric syndrome [9].

The protective roles of the Mediterranean diet, Dietary Approaches to Stop Hypertension (DASH) diet, and Mediterranean-DASH Intervention for Neurodegenerative Delay (MIND) diet in several geriatric syndromes have been documented in recent years [10,11]. Data-mining the “Nutrition and Health Survey in Taiwan” database, our previous studies have discovered protective dietary patterns for geriatric syndromes, such as frailty and poor cognitive function [12,13]. This protective Taiwanese eating approach (TEA) is similar to the Mediterranean and DASH diets, but with some local features; it is enriched with nutrient and phytonutrient-rich plant foods (vegetables, fruits, whole grains, and nuts and seeds) and drinks (tea), fish, shellfish, and other protein-rich foods, such as eggs and milk, but less white rice or noodles [9]. This eating approach is also in line with the Taiwanese Daily Food Guide, which emphasizes vegetables, fruits, nuts and seeds, whole grains, dairy, and other protein-rich foods in the order of soybean products, fish/seafood, eggs, and meat [14]. Tea drinking is a unique protective component in this heathy TEA dietary pattern. In addition, previous randomized controlled trials that we conducted in Miaoli Hospital, Taiwan, have shown that the frailty score may be significantly reduced after a dietary intervention, which informed the individualized energy intake level, coupled with the recommendation on the balanced six food groups, according to the Taiwanese Daily Food Guide, while at the same time providing one daily serving of skim milk powder and one serving of nuts and seeds [15,16].

Although it is well recognized for frailty management that combining a nutrition program with physical exercise has produced a better outcome than each individual component alone [17], and social participation may exert a synergistic effect [18], nutrition intervention programs featuring fun group activities are scarce. Therefore, we recruited 13 community caring strongholds in the metropolitan Taipei area and carried out a single-blind, randomized, controlled trial to investigate the efficacy of a series of diet and nutrition group activities (once a week, lasting for 3 months) in changing the dietary intakes of six food groups, the frailty status, and working memory.

## 2. Methods

### 2.1. Ethics and Trial Registration

The study protocol and informed consent form was reviewed and approved by the Institutional Review Board on Biomedical Science Research of Academia Sinica in Taiwan (AS-IRB01-16057). The trial was retrospectively registered on 28 October 2021 at www.clinicaltrials.gov as NCT05129163.

### 2.2. Participants

The research team downloaded a list of Geriatric Community Caring Strongholds from the official website of the Department of Social Welfare of the Taipei City government. Those centers that provided lunch at least twice a week for older adults were contacted for recruitment. The research team then provided a briefing to each of those willing community strongholds. Once they agreed, recruitment and activities were carried out center by center from August 2018 to May 2019. Volunteers were excluded if they had any of the following conditions: communication disability, severe disease (e.g., cancer, chronic kidney disease, etc.), dietary control by doctors’ instructions, or inability to walk 14 m independently. All subjects signed the informed consent form before their participation.

### 2.3. Sample Size

**Figure 1:**
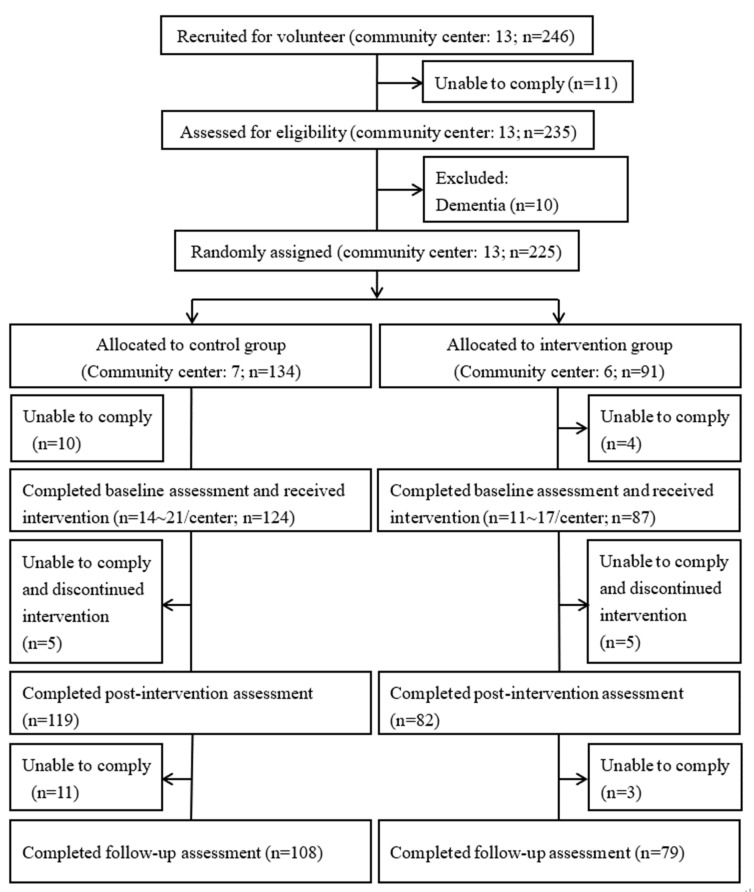
Flow of recruitment.

Our previous one-to-one intervention study reported significant effects of an individualized nutrition education with customized dishware on the frailty score [15]. The mean change and standard deviation of the frailty score in this study were applied to estimate the sample size, with the cluster size set at 15 and the intra-cluster correlation coefficient set at 0.05 [19]. Therefore, 101 subjects from 7 community centers for each group are required to detect a significant effect of improved nutrition on the frailty score with a 2-sided significance level of 5% and a power of 90%. We aimed to recruit 225 subjects from 14 community centers to allow for a 10% dropout. Figure 1 shows that a total of 235 volunteers were screened. We recruited at 14 community centers. Among them, the last one declined due to the outbreak of the coronavirus disease 2019. Among the remaining 13, a total of 225 subjects were eligible to participate in the study, and 211 subjects completed the baseline assessment and received intervention. The number of subjects in the community centers were 18, 20, 21,14, 21, 14, and 16 for the control group, and 11, 15, 14, 17, 17, and 13 for the intervention group.

### 2.4. Study Design

The study was a cluster-randomized, single-blind, and parallel controlled trial that consisted of a 3-month treatment period and another 3-month follow-up. According to the cluster size and location of the community centers, each community center was randomly allocated to either the control or the intervention group. Participants in the same community centers were allocated to the same treatment, so that they were masked to the treatment allocation. The random assignment of community centers to treatment groups was performed with Minim software (version 1.5; London Hospital Medical College) [20]. Both groups received weekly one-hour exercise in the treatment period under the supervision of New Taipei City “Exercise is Medicine^®^” certified coaches. Exercises designed for older adults included aerobic exercise, muscle strength training, balance and coordination exercise, and stretching. The nutrition group received additional weekly one-hour nutrition education led by licensed dieticians, which featured a 10-min short talk and a 50-min group activity designed by this research team.

### 2.5. Dietary Intervention

The aim of the nutritional education was to promote the Taiwanese Daily Food Guide for elderlies in everyday practice [14], supplemented with the TEA food choice principle associated with geriatric syndromes. Each participant was encouraged to consume the correct servings of the six food groups at his or her individualized energy level; to select diverse nutrient-dense foods from each food group; to prioritize protein foods (soy and beans, aquatic foods, eggs, poultry, and meat, in that order); and to drink a cup of tea, coffee, or caffeine-free herbal tea every day. We designed five key-concept courses. In the first 5 weeks of intervention, participants received intervention via group activities on ① know my plate and food groups, ② whole grains and roots, ③ drinking teas with dairy, and nuts and seeds, ④ novel ways to eat fruits and vegetables, and ⑤ healthy breakfast ideas. Several tools, such as WAKE Taiwan website (https://healthybody.nhri.org.tw/Portal/foodsuggest.aspx (accessed during 27 August 2018 to 4 June 2019)) for estimating energy levels, food models, and three-dimensional dining plate designed by the Taiwanese Association of Diabetes Educators [21], were employed in the teaching.

We designed a bookmark with three plate images (each for one dining meal), which corresponds to the three-dimensional dining plate, in order for older adults to avoid memorizing the amount of foods. In the first week, among other things, the dietitians guided each participant to color (green for vegetables, red for protein-rich foods, brown for oil and nuts, gray for rice, and blue for milk) the designated spaces on the bookmark for each food group, corresponding to the amount he or she should ideally consume. Moreover, by adopting the concept of Bloom’s Taxonomy [22], the Trans-theoretical Model (including the Stages of Change Model) [23], and information processing [24], we designed group activity sessions for weeks 2–5, which contained the cognitive domain (knowledge), affective domain (attitude), and psychomotor domain (practical methods), on nutrition and health using “fun elements” incorporated into group activities, such as group competitions, Bingo, matching games, and category games. These above-mentioned multi-domains and fun elements were intended to stimulate the progression of individuals’ stage of behavior change. As a matter of fact, every participant at different stages of behavior change gained benefits from the group level of activity sessions.

In week 6, we provided individual reports about their dietary intake and frailty status assessed at baseline to motivate our participants to comply with the intervention. During week 7 and week 8, participants were asked to take one-day photos of their diet for conducting a photo-elicitation focus group. In the sessions, we used anonymized photos that participants had shared to elicit a discussion on how the presented diet might be changed in order to comply with participant’s energy requirement and targeted serving amounts of the six food groups. In the last 4 weeks of intervention, targeting the same themes as week 2 to week 5, we designed practice activities that helped to break down barriers we observed towards establishing long-term changes in dietary habits. Examples of the practice activities include tasting buns and bread made with whole grains, blindly comparing whole milk and low-fat milk, making an afternoon tea set containing a cup of tea with milk powder and nuts, and weighing out a portion of vegetables from commercially bagged vegetables and blanching it. We also encouraged participants to share how they achieved the Daily Food Guide in their own creative ways. 

In addition to the nutritional group activity sessions, building up the supporting environment for frailty prevention in the community center was executed. Before the intervention, we trained onsite social workers and staff to estimate participants’ energy requirements, and designed a standard operation procedure to train them for serving proper amounts of foods to individuals based on their needs. They also received training on menu planning (food diversity), food preparation (food cutting and softening), healthy cooking methods (using herbs and tasty foods in dish to replace deep frying and heavy sauces), and techniques of motivational interviews focused on geriatric syndrome prevention and control.

### 2.6. Data Collection

Once the volunteers signed the informed consent, arrangements were made for an interview on their socio-demographics, self-reported disease history, and medication. An assessment was made on their diet, frailty, working memory, performance on a number cancellation task, and depression. The assessment was repeated at the post-intervention phase (3 months) and 6-month follow-up.

### 2.7. Frailty Assessments

The frailty status was defined using modified Linda Fried criteria with Taiwanese cutoff points [15,16]. The detailed description of the operating procedures was as previously published [15,16]. Moreover, an advanced hand dynamometer, the JTECH Commander Echo Grip tester (JTECH Medical Industries, Inc., Midvale, UT, USA), was applied for handgrip measurements in this study.

### 2.8. Working Memory and Depression Assessment

Both the forward and backward digit-span tasks of the Wechsler intelligence scale [25], and the number cancellation task of the Alzheimer’s Disease Assessment Scale [26] were used to assess working memory. The Geriatric Depression Scale-Short Form (GDS-SF), Chinese version, a 15-item assessment tool, was used to identify depression in people 65 years old or older [27,28]. Participants were classified by their point scores into ‘normal’ (for 0 to 4 points), ‘at risk of depression’ (for 5 to 9 points), and ‘in a state of depression’ (for ≥10 points).

### 2.9. Dietary Assessment

According to the food frequency questionnaire from the Nutrition and Health Survey in Taiwan, a modified version was developed based on the most frequently consumed food groups and applied for the dietary assessment [29]. It consisted of three parts: the first was the frequency of 18 food groups (estimated with age and sex-specific average portion size), the second was the consumption frequency and amount of 10 food groups with typical portion sizes, and the last was the dining habits for estimating types and amounts of cooking oil and salts. The assessment allowed estimating the number of servings that participants consumed from each of the six food groups in the past month. The six food groups, as defined by the Health Promotion Agency in Taiwan, were ① total grains and roots (sub-grouped as non-refined and refined); ② legumes, aquatic protein foods, eggs, and meats; ③ dairy; ④ fruits; ⑤ vegetables; and ⑥ oils (sub-grouped as cooking oils and as nuts and seeds) [30].

### 2.10. Assessment of Stages in Practicing Healthy Dietary Behaviors

The five stages of behavior change include pre-contemplation, contemplation, preparation, action, and maintenance [23]. The stage of consuming an adequate serving of each of the six food groups was determined for each participant in the experimental group. Taking dairy as an example, the pre-contemplation stage was defined as when the subject did not consume a daily 1.5 serving of dairy (e.g., 360 mL milk per day) and had no intention to change within 6 months; the contemplation or the preparation stage was defined as when the subject did not meet the daily intake recommendation, but intended to or was preparing to take action; and the action and maintenance stages were defined as when the subject met the daily intake recommendation within and longer than 6 months, respectively. Although the intervention period lasted for 3 months only, the maintenance stage possibly occurred if the subject followed the recommendation before the intervention. Stage-of-change data were collected immediately after the group nutrition activity session on the activity day in week 2 to week 4 (as baseline). The data collection was repeated again in week 9 to week 11 (between 2 to 3 months of intervention).

### 2.11. Data Management and Statistical Analysis

Answers to the questions were recorded by ballpoint pen on the questionnaire, which were later recognized by an optical mark recognition system (Alldala Global Service Co., Ltd., Zhubei City, Hsinchu County, Taiwan). Raw data were carefully cleaned before analysis. For dietary data, the average total energy intakes and the number of servings from each of the six food groups were estimated; they were further adjusted to the 1500 Kcal level by the density method [31]. 

The homogeneity of the clusters was examined for multiple baseline characteristic variables. There were no statistical differences found for age, frailty status, and self-reported chronic diseases. Modest differences were observed for male proportion, education level, and caloric intake.

The frailty component score (0 to 5), frailty status (0 was robust, 1 was prefrail, and 2 was frail), and dietary intakes were the primary outcomes. The secondary outcomes included working memory and geriatric depression. The intention-to-treat principle was applied to replace all post-intervention missing values with their baseline data. Baseline characteristics were compared between the control and the experimental group using the Student’s *t*-test for continuous variables and either the chi-square test or Fisher’s exact test for categorical variables. The linear mixed model was applied for comparisons of the intervention effects over time on measured outcomes. In this model, the outcomes of interest were responsible variables; treatment (0, 1), time (0 for baseline, 1 for either 3-month post-intervention or 6-month follow-up), and treatment × time interaction were explanatory variables. Even though educational level could affect one’s diet, this study focused on the dietary changes before and after the intervention, and the changes in outcome of interest were confirmed to be similar, regardless of educational level. Therefore, educational level was not adjusted in the model. In addition, an autoregressive correlation structure was used. The statistical significance levels of all tests were set at 0.05. All statistical analysis was performed using SAS version 9.4 (SAS Institute, Cary, NC, USA).

For stage-of-change results, participants in the experimental group were classified into subgroups according to their status shift in the healthy dietary behavioral stages, including the improved, maintained, and deteriorated subgroups. Those who improved in their stages of behavior change and those who remained at the stage of either action or maintenance were all classified in the “improved or maintained at advanced stages” subgroup. Those who moved their stages of behavior change back to a former stage and those who remained at the stage of pre-contemplation, contemplation, or preparation were classified in the “deteriorated or maintained at early stages” subgroup. The dietary intakes and health parameters were compared between these two groups; no such data were available for the control group for analysis.

## 3. Results

Among the 211 participants, 201 participated in the intervention and completed post-intervention measurement, and 187 further completed the 6-month follow-up measurement (Figure 1). During the intervention period, the attendance rates of the control and the nutrition group were 86.8% and 79.8%, respectively.

### 3.1. Baseline Characteristics

The baseline characteristics and habitual dietary intakes of the participants are presented in Table 1 and Table 2, respectively. The study population was primarily women (76.3%) with some self-reported comorbidities, mean age at mid-70th, and mean body mass index (BMI) near 24. The sex distribution, self-reported comorbidity, GDS-SF score, and distribution of frailty components were not significantly different between the two groups. However, the nutrition group was significantly older (by 2 years), with fewer participants who had been educated for more than 6 years (by 17%), and with higher mean BMI values (by 1 unit), poorer working memories (both forward and reverse digit span by less than 1 digit), and lowered number cancellation task scores (by 3 scores). However, the differences of magnitude are small in general.

The mean energy intake was 1368 ± 26.5 Kcal, and the distributions of energy from carbohydrates, proteins, and fat were 57.6%, 14.7%, and 27.7%, respectively, with no significant difference between the two groups. In terms of the six food groups, significantly lower intakes of non-refined grains and roots (by 0.25 bowls) and dairy (by 0.13 servings) were observed in the nutrition group.

### 3.2. Effect of Nutrition Education Program on Dietary Intakes

**Figure 2:**
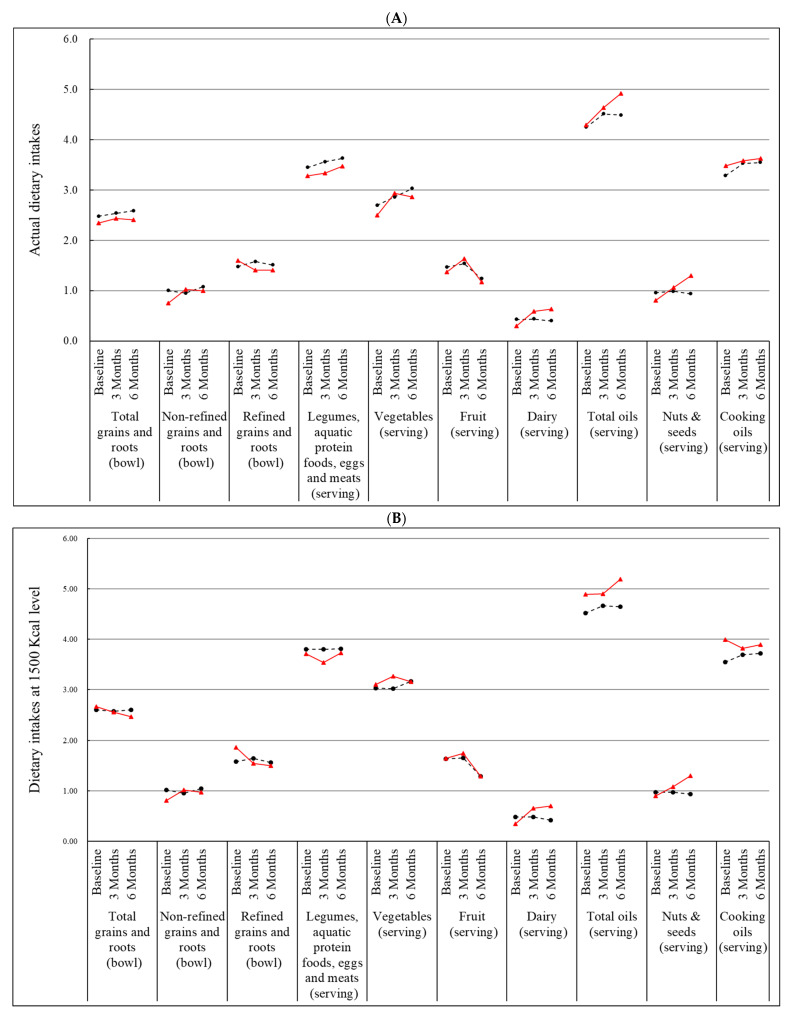
Effect of nutrition education program on dietary intakes. Data are mean change from baseline; *n* = 124 at 3 months and 108 at 6 months for Control group (black lines), and 87 at 3 months and 79 at 6 months for Nutrition group (red lines). (**A**) Actual dietary intake; (**B**) Dietary intake on the basis of 1500 Kcal.

Figure 2 depicts the trends of dietary intakes from baseline to the end of follow-up. The results of the intervention effects on dietary intakes are summarized in Table 3, as indicated by the time × treatment effect, with treatment and time variables also in the model. The trends of the dietary intake changes of total grains and roots were not different between the treatment groups at both 3 months and 6 months. When looking into the grains and roots quality, the nutrition group, compared with the control, had a significantly greater increase in non-refined grains and roots (*p* = 0.003), and at the same time, a greater reduction in refined grains and roots (*p* = 0.008) at 3 months, but the effects were no longer significant at the 6-month follow-up. The experimental group also had a significantly larger increase in dairy intake than the control at both the post-intervention (*p* < 0.0001) and the follow-up (*p* < 0.0001). In addition, the experimental group tended to have increased intake of the ‘soybean products, aquatic protein foods, eggs, and meats’ group, vegetables, fruit, oils, ‘nuts and seeds’ group, and ‘cooking oils’ subgroup, although these increases were not significantly different between the two arms at both 3 months and 6 months. The increase in nuts and seeds of the experimental group tended to be larger compared to the control post-intervention (*p* = 0.080) and at follow-up (*p* = 0.002). Similar results were found after the caloric adjustment to 1500 Kcal.

### 3.3. Effect of Nutrition Education Program on Frailty Status and Frailty Component Score

The results of the effects of the nutrition education program on the frailty score and frailty components are presented in Table 4. The frailty status regression was significantly greater for the nutrition group compared to the control at the post-intervention phase (*p* = 0.036), but this phenomenon disappeared at follow-up. The grip strength seemed to increase more in the experimental group than the control group at both the post-intervention phase (*p* = 0.077) and the follow-up (*p* = 0.056). None of the other frailty components (weight, 10 m walking time, and physical activity) were significantly affected by the interventions.

### 3.4. Effect of Nutrition Education Activity on GDS-SF and Working Memory

The results on the effects of the nutrition education program on GDS-SF, working memory, and the number cancellation task are presented in Table 5. No statistically significant intervention effect was observed regarding GDS-SF. As for working memory, the intervention improved the forward digit span performance more for the nutrition group than the control group at 3 months (*p* = 0.004) and at the 6-month follow-up (*p* = 0.007). However, the intervention did not affect working memory in the reverse digit span and the number cancellation task.

### 3.5. Effect of Nutrition Education Program on Self-Reported Stages of Healthy Dietary Behaviors

**Figure 3:**
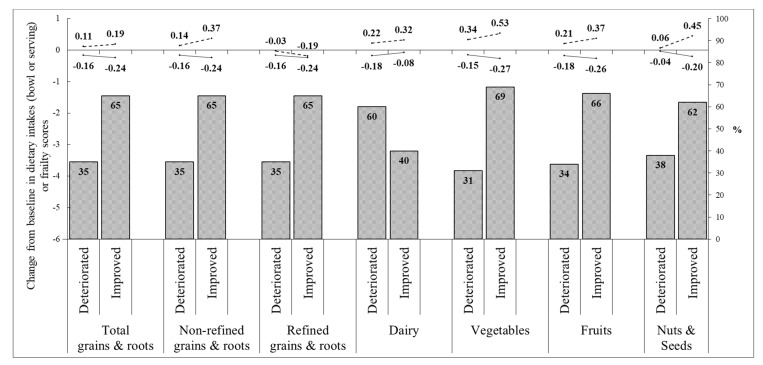
Effects of nutrition program on changes of dietary behavioral stages, dietary intakes, and frailty scores. The grey bars at the bottom represent “the improved or maintained at advanced stages” subgroup and “the deteriorated or maintained at early stages” subgroup, classified by the status of nutrition group participants’ shift in each healthy dietary behavioral stage at post-intervention. According to the two subgroups for each healthy dietary behavior, the changes from baseline in dietary intakes (···) and frailty scores (―) are shown in lines at the top of the figure.

Since the control group members were not given a nutrition education session, we did not have the opportunity to collect information on their health behaviors. In the sub-analysis of the nutrition group, with regards to non-refined grains and roots, dairy, vegetables, fruits, and nuts and seeds, respectively, we found 65%, 40%, 69%, 66%, and 62% of the participants advanced in the behavior change stage defined by the “Stage of Change Model” (Figure 3). Among those with improved stages for non-refined grains and roots, vegetables, fruits, and nuts and seeds, their dietary intakes, as well as the frailty scores, were all improved more than their deteriorated counterpart.

## 4. Discussion

In this cluster-randomized controlled trial, we randomized 13 community strongholds into 2 treatment groups—exercise alone (control group) and exercise and nutrition combined (experimental group)—conducting a 3-month intervention using group activity sessions. This study assessed whether adding a dietary intervention on top of an exercise session could regress the development of frailty and related geriatric parameters via favorable dietary intake changes. The results showed that the 3-month intervention significantly improved the frailty status, as well as working memory in the forward digit span, along with the improvement in the dietary intakes of non-refined grains and roots, dairy products, and nuts and seeds (at borderline). The dietary improvements in the experimental group were also accompanied by more advanced dietary behavior change stages. However, the beneficial effects only persisted for the forward digit span and dietary intake of dairy products and nuts and seeds, not for the frailty status, which suggests that continuous boosting activities might be needed for a long-term maintenance effect.

The literature has demonstrated the effects of nutrition and/or physical activity treatment on physical frailty status [32,33,34,35,36]. Physical training normally has positive effects on physical performance. As for nutritional intervention, the beneficial effects of daily protein supplements containing about 30 g protein, in addition to the normal diet, are well established for frailty prevention [37,38]. On the other hand, the literature on the effectiveness of other oral supplements, such as vitamin D [39,40,41], omega-3 fatty acids [42,43], and mixed nutrients [15,35], on frailty is limited. Instead of providing specific nutrients, several studies intervened with dietary counseling [15,16,36,44,45]; however, the effects on frailty prevention were inconclusive [46]. The present group intervention study has shown that the beneficial effects were accompanied by the increased intakes of the non-refined ‘grains and roots’ subgroups, dairy products, and ‘nuts and seeds’ and the reduced intake of refined grains. Thus, the beneficial effects might presumably be due to the increased consumption of energy, protein, and other micronutrients in these foods or their interactions. The effect of dietary intervention we demonstrated is one that was administered alongside the simultaneous exercise session; it is not clear whether nutrition intervention alone would have the same effect.

Most intervention studies targeting frailty prevention were designed at the individual level—in which participants took their oral supplements, performed physical exercise, or were counseled by dieticians—whereas very few of them were at the group level [38,47]. A Japanese study demonstrated that 12-week group training classes including physical exercise training and nutritional programs (EN group) were more effective than the exercise group and the control group (neither exercise nor a nutrition program) on the quality of life (role emotional score) in prefrail elderly women living in communities [38]. However, for physical frailty phenotypes, no significant changes in handgrip strength and walking speed were observed within the EN group, nor was a difference found among the three groups. The similarities between the Japanese study and the present study included the cluster size (about 15 participants per session), the frequency and the duration of physical training, and a short talk before nutritional activities. Some discrepancies between the two studies were the goal and the duration of the nutritional intervention. The Japanese study focused on practical sessions using food ingredients rich in protein and vitamin D for cooking. For each participant, a typical meal with side dishes contained 350–400 Kcal, 20–22 g protein, and 5–10 µg vitamin D, not counting staple foods. In the present study, the energy level was targeted at the individual level, so the goals for protein foods and other food groups were varied and attended to individually. In addition, fun group activities were conducted instead of cooking classes. There is another ongoing project in Malaysia assessing the effectiveness of group nutrition education and exercise to prevent frailty [47]. The efficacy of this intervention at group levels is worthy of our attention.

Intervention through group activity sessions might have induced some peer effects in individual participants [48]. Eating lunch together is a form of social participation. Our participants might have been affected by their peers, onsite working staff, the personalized lunch, and group activities. In addition, our study employed multiple theories and approaches. In this complex situation, it is hard to attribute each effect to any individual component; thus, we deem multiple strategies essential to the success of frailty management.

This study had several limitations. First of all, the participants in the present study were able to walk 14-m independently, indicating that they were likely better than average in physical functioning. In fact, the majority of the participants were at a robust and pre-frail status, and only about 5% of them were frail at baseline. Therefore, we cannot extrapolate our findings to elders with severe frailty. Second, no health behavior data were available to assess the stages of behavior change for the control group. This information should be collected in the future for making firm conclusions on the relationships among changes of the behavioral stage and changes of the dietary and health outcomes.

## 5. Conclusions

In conclusion, nutrition group activities in community centers are likely to promote healthy eating in elders, which may in turn regress their frailty status and improve their working memory. The effect of this group-level intervention is comparable to or better than that of the individual-level ones, while the manpower used is much less. We found that physical functional changes were accompanied by changes in dietary intake. However, the improved frailty status backslid after the intervention ceased, suggesting that boosting activities or maintenance mechanisms might be needed to prevent waning of the intervention effect.

## Figures and Tables

**Table 1:** Baseline characteristics of subjects ^1^.

	Control (*n* = 124)	Nutrition (*n* = 84–87)	*p* Values ^a^
Age (years)	73.1 ± 0.60	75.23 ± 0.69	0.021
Sex (females)	98 (79.03)	63 (72.41)	0.266
Education (>6 years)	95 (76.61)	52 (59.77)	0.009
Weight (Kg)	57.28 ± 0.82	59.38 ± 1.18	0.133
Body mass index (kg/m^2^)	23.54 ± 0.30	24.56 ± 0.42	0.044
Self-reported comorbidity			
Hypertension	64 (51.61)	43 (49.43)	0.754
Diabetes mellitus	25 (20.16)	24 (27.59)	0.209
Myocardial infraction	6 (4.84)	4 (4.60)	1.000
Stroke	5 (4.03)	4 (4.60)	1.000
Cancer	11 (8.87)	7 (8.05)	0.833
Geriatric Depression Scale (score)	1.37 ± 0.21	1.24 ± 0.18	0.646
Working memory			
Forward Digit Span (digit)	7.83 ± 0.12	6.95 ± 0.17	<0.0001
Reverse Digit Span (digit)	4.44 ± 0.13	3.94 ± 0.15	0.012
Number Cancellation Task (score)	19.91 ± 0.8	16.86 ± 0.92	0.014
Frailty score	0.57 ± 0.07	0.66 ± 0.09	0.481
Frailty components			
Unintentional weight loss	12 (9.68)	10 (11.49)	0.671
Self-reported exhaustion	19 (15.32)	13 (14.94)	0.940
Weak grip strength	30 (24.19)	22 (25.29)	0.856
Slow gait speed	3 (2.42)	5 (5.75)	0.279
Low level of physical activity	7 (5.65)	8 (9.20)	0.416
Maximum grip strength of dominant hand (Kg)	21.32 ± 0.58	21.18 ± 0.71	0.879
10 m walking time (second)	9.08 ± 0.22	9.27 ± 0.46	0.711
Physical activity (Kcal/week)	2691 ± 149.6	2636 ± 241.1	0.846

^1^ Data are presented as mean ± SEMs or *n* (%). ^a^ Student’s *t*-test or the Chi-square test or Fisher’s exact test were used to compare parameters of interest between the treatment groups for continuous and categorical variables, respectively.

**Table 2:** Baseline daily dietary intakes of the participants by treatment groups ^1^.

	Control (*n* = 124)	Nutrition (*n* = 87)	*p* Values ^a^
Energy intake (Kcal)	1405 ± 36	1315 ± 38	0.092
Protein (% calories)	14.87 ± 0.21	14.4 ± 0.25	0.147
Fat (% calories)	27.49 ± 0.51	28.04 ± 0.70	0.522
Carbohydrate (% calories)	57.64 ± 0.55	57.57 ± 0.80	0.942
Total grains and roots (bowl)	2.48 ± 0.09	2.35 ± 0.10	0.369
Non-refined (bowl)	1.00 ± 0.07	0.75 ± 0.08	0.024
Refined (bowl)	1.48 ± 0.08	1.60 ± 0.09	0.300
Legumes, aquatic protein foods, eggs, and meats (serving)	3.45 ± 0.12	3.28 ± 0.15	0.344
Dairy (serving)	0.43 ± 0.05	0.30 ± 0.04	0.034
Vegetables (serving)	2.70 ± 0.14	2.50 ± 0.15	0.327
Fruits (serving)	1.47 ± 0.08	1.37 ± 0.10	0.418
Total oils (serving)	4.25 ± 0.20	4.29 ± 0.22	0.904
Nuts and seeds (serving)	0.96 ± 0.12	0.81 ± 0.10	0.327
Cooking oils (serving)	3.29 ± 0.15	3.48 ± 0.20	0.445

^1^ Data are presented as mean ± SEM. ^a^ Independent sample *t*-test used to compare the difference between groups.

**Table 3:** Effect of nutrition education program on dietary intakes ^1^.

	3 Months			6 Months		
	Control (*n* = 124)	Nutrition (*n* = 87)	*p* ^a^	Control (*n* = 108)	Nutrition (*n* = 79)	*p* ^a^
*Actual dietary intakes*						
Total grains and roots (bowl)	0.06 ± 0.09	0.09 ± 0.09	0.792	0.09 ± 0.08	0.06 ± 0.11	0.758
Non-refined (bowl)	−0.05 ± 0.07	0.28 ± 0.08	0.003	0.05 ± 0.09	0.22 ± 0.11	0.190
Refined (bowl)	0.11 ± 0.07	−0.19 ± 0.09	0.008	0.03 ± 0.08	−0.16 ± 0.10	0.087
Legumes, aquatic protein foods, eggs and meats (serving)	0.10 ± 0.11	0.06 ± 0.15	0.824	0.16 ± 0.13	0.21 ± 0.13	0.840
Vegetables (serving)	0.15 ± 0.15	0.44 ± 0.17	0.214	0.27 ± 0.14	0.42 ± 0.17	0.657
Fruit (serving)	0.06 ± 0.09	0.27 ± 0.10	0.132	−0.22 ± 0.09	−0.16 ± 0.11	0.739
Dairy (serving)	0 ± 0.04	0.29 ± 0.05	<0.0001	−0.05 ± 0.04	0.33 ± 0.05	<0.0001
Total oil (serving)	0.26 ± 0.20	0.35 ± 0.21	0.766	0.22 ± 0.21	0.65 ± 0.22	0.179
Nuts and seeds (serving)	0.02 ± 0.08	0.25 ± 0.11	0.080	−0.02 ± 0.08	0.47 ± 0.16	0.002
Cooking oil (serving)	0.24 ± 0.17	0.10 ± 0.18	0.583	0.24 ± 0.19	0.17 ± 0.19	0.741
*Dietary intakes on the basis of 1500 Kcal*						
Total wholegrains (bowl)	−0.01 ± 0.05	−0.11 ± 0.06	0.205	0.02 ± 0.05	−0.19 ± 0.06	0.008
Non-refined wholegrains (bowl)	−0.07 ± 0.06	0.21 ± 0.07	0.003	0.03 ± 0.07	0.13 ± 0.09	0.276
Refined wholegrains (bowl)	0.06 ± 0.06	−0.32 ± 0.08	<0.0001	−0.01 ± 0.07	−0.33 ± 0.10	0.005
Soybean, fish, eggs, and meat (serving)	0 ± 0.12	−0.17 ± 0.14	0.349	0.02 ± 0.12	0.04 ± 0.13	0.946
Vegetables (serving)	0 ± 0.16	0.17 ± 0.21	0.493	0.09 ± 0.15	0.12 ± 0.21	0.949
Fruit (serving)	0.02 ± 0.09	0.10 ± 0.11	0.541	−0.31 ± 0.09	−0.32 ± 0.12	0.950
Dairy (serving)	0 ± 0.04	0.31 ± 0.06	<0.0001	−0.07 ± 0.04	0.35 ± 0.06	<0.0001
Total oil (serving)	0.14 ± 0.18	0.01 ± 0.24	0.677	0.18 ± 0.20	0.29 ± 0.26	0.617
Nuts and seeds (serving)	0 ± 0.09	0.18 ± 0.11	0.191	−0.02 ± 0.09	0.38 ± 0.16	0.012
Cooking oil (serving)	0.14 ± 0.16	−0.17 ± 0.22	0.243	0.20 ± 0.18	−0.09 ± 0.25	0.359

^1^ Values are mean changes ± SEMs from baseline. ^a^ Linear mixed model analysis was undertaken on the outcome variable, with the treatment (0, 1), time (0 for baseline, 1 for either 3-month post-intervention or 6-month follow-up), and treatment × time interaction as fixed effects and subjects as random effects. The presenting *p* values were the interaction effect.

**Table 4:** Effect of nutrition education program on frailty scores and frailty components ^1^.

	3 Months			6 Months		
	Control	Nutrition	*p* ^a^	Control	Nutrition	*p* ^a^
Frailty score	−0.03 ± 0.06	−0.2 ± 0.08	0.101	−0.10 ± 0.07	−0.08 ± 0.10	0.652
Frailty status	−0.01 ± 0.05	−0.16 ± 0.06	0.036	−0.02 ± 0.05	−0.06 ± 0.07	0.806
Weight (Kg)	0.08 ± 0.18	0.45 ± 0.18	0.158	0.34 ± 0.2	0.43 ± 0.25	0.819
Grip strength (Kg)	−0.27 ± 0.19	0.29 ± 0.26	0.077	−0.47 ± 0.23	0.21 ± 0.26	0.056
Walking for 10 m (second)	0.11 ± 0.13	−0.18 ± 0.16	0.146	−0.26 ± 0.16	−0.38 ± 0.17	0.564
Physical activity (Kcal/week)	840.7 ± 144.4	722.4 ± 233.4	0.649	724.5 ± 154.5	1106 ± 244.6	0.163

^1^ Values are mean changes ± SEMs from baseline. ^a^ Linear mixed model analysis was undertaken on the outcome variable, with the treatment (0, 1), time (0 for baseline, 1 for either 3-month post-intervention or 6-month follow-up), and treatment × time interaction as fixed effects and subjects as random effects. The presenting *p* values were the interaction effect.

**Table 5:** Effect of nutrition education program on Geriatric Depression Scale and working memory ^1^.

	3 Months			6 Months		
	Control	Nutrition	*p* ^a^	Control	Nutrition	*p* ^a^
Geriatric Depression Scale (score)	0.22 ± 0.15	0.06 ± 0.18	0.502	−0.23 ± 0.18	0.03 ± 0.20	0.306
Working memory						
Forward Digit Span	0.42 ± 0.09	0.85 ± 0.12	0.004	0.37 ± 0.09	0.81 ± 0.14	0.007
Reverse Digit Span	0.53 ± 0.10	0.48 ± 0.11	0.698	0.68 ± 0.11	0.55 ± 0.12	0.325
Number Cancellation Task	−0.63 ± 0.59	0.14 ± 0.80	0.460	−0.69 ± 0.69	−0.01 ± 0.85	0.619

^1^ Values are mean changes ± SEMs from baseline. ^a^ Linear mixed model analysis was undertaken on the outcome variable, with the treatment (0, 1), time (0 for baseline, 1 for either 3-month post-intervention or 6-month follow-up), and treatment × time interaction as fixed effects and subjects as random effects. The presenting *p* values were the interaction effect.

## Data Availability

The datasets used in the current study are not publicly available due to the legal restrictions of the Personal Information Protection Act legislated by the government of Taiwan. Data are available from the corresponding authors upon reasonable request with valid proposals and a confidentiality agreement.

## Abbreviations

DASH: Dietary Approaches to Stop Hypertension; BMI: body mass index; EN group: physical exercise training and nutritional program group; GDS-SF: Geriatric Depression Scale-Short Form; MIND: Mediterranean-DASH Intervention for Neurodegenerative Delay; TEA: Taiwanese eating approach.
